# Study of the Properties of Bearberry Leaf Extract as a Natural Antioxidant in Model Foods

**DOI:** 10.3390/antiox5020011

**Published:** 2016-04-01

**Authors:** Nurul Aini Mohd Azman, Maria Gabriela Gallego, Francisco Segovia, Sureena Abdullah, Shalyda Md Shaarani, María Pilar Almajano Pablos

**Affiliations:** 1 Chemical Engineering Department, Technical University of Catalonia, Avinguda Diagonal 647, Barcelona 08028, Spain; ainiazman@gmail.com (N.A.M.A.); maria.gabriela.gallego@upc.edu (M.G.G.); segoviafj@gmail.com (F.S.); 2 Chemical and Natural Resources Engineering Faculty, University Malaysia Pahang, Lebuhraya Tun Razak, Pahang 26300, Malaysia; sureena@ump.edu.my (S.A.); shalyda@ump.edu.my (S.M.S.)

**Keywords:** bearberry leaves, scavenging activity, lipid oxidation, active packaging film, antioxidant activity

## Abstract

The common bearberry (*Arctostaphylos uva-ursi* L. Sprengel) is a ubiquitous procumbent evergreen shrub located throughout North America, Asia, and Europe. The fruits are almost tasteless but the plant contains a high concentration of active ingredients. The antioxidant activity of bearberry leaf extract in the 2,2′-azino-bis-3-ethylbenzothiazoline-6-sulphonic acid (ABTS) radical cation assay was 90.42 mmol Trolox equivalents/g dry weight (DW). The scavenging ability of the methanol extract of bearberry leaves against methoxy radicals generated in the Fenton reaction was measured via electron paramagnetic resonance. Lipid oxidation was retarded in an oil–water emulsion by adding 1 g/kg lyophilised bearberry leaf extract. Also, 1 g/kg of lyophilised bearberry leaf extract incorporated into a gelatin-based film displayed high antioxidant activity to retard the degradation of lipids in muscle foods. The present results indicate the potential of bearberry leaf extract for use as a natural food antioxidant.

## 1. Introduction

Lipid oxidation in food causes serious problems that lead to short shelf lives and loss of nutritional quality [1]. Synthetic antioxidants such as butylated hydroxyanisole (BHA), butylated hydroxytoluene (BHT), and tert-butylhydroquinone (TBHQ) have been used as antioxidants in many food products [2], but consumers have become concerned about possible toxicological effects and often prefer natural antioxidants for foods consumed as part of a healthy diet. Thus, many investigations have focused on identification of novel antioxidants to be tested in model foods such as emulsions and incorporated into packaging films.

Natural antioxidants contain a high concentration of phenolic compounds and normally occur in fruits, vegetables, and herbs [3,4]. Bearberry (*Arctostaphylos uva-ursi* L. Sprengel) is a ubiquitous procumbent evergreen shrub located throughout North America, Asia, and Europe. The fruits are almost tasteless despite containing a high concentration of active ingredients in many commercial products [5]. The antioxidant potential of bearberry leaves (BL) has been studied by numerous chemical assays including reducing power assay, radical scavenging activity using 2,2-diphenyl-1-picrylhydrazyl (DPPH), liposome model, scavenging hydroxyl radicals (HO), and linoleic acid model system [6,7]. The main constituents of BL are the glycosides arbutin (5%–15%), methylarbutin (up to 4%), and small quantities of the free aglycones. Other constituents include ursolic acid, tannic acid, gallic acid, *p*-coumaric acid, syringic acid, galloylarbutin, gallo-tannins, and flavonoids, notably glycosides of quercetin, kaempferol, and myricetin [8]. The traces of polyphenols in BL have made them promising candidates as potential protectors against lipid oxidation and biological ageing of tissues.

Additionally, different phenolic compounds may act as antioxidants with varying efficiency in different food systems, which depend on their polarity and molecular characteristics. Studies on the efficacy of natural polyphenol derived from plants in food models are not new. Natural phenolics identified in many fruits, herbs, and vegetables used as antioxidants that retard the oxidative rancidity of foods in numerous food applications were summarized by Kiokias *et al.* [9]. They also critically discussed the recent new technology using multiple emulsions along with innovative applications of natural antioxidants in food [10]. Several studies of the antioxidant activity of BL at several concentrations have been conducted in a meat model [11,12] and these have successfully demonstrated the potential of bearberry to inhibit the degradation of lipids in pork. The potential of incorporating plants with phenolic compounds in emulsion was critically assessed by Kiokias *et al.* [13]. Among the tested flavonoid compounds, quercertin significantly reduced the oxidative deterioration of cottonseed oil-in-water emulsions. Quercetin is one of the phenolic compounds found in the BL. Hence, the potential effects of BL extract in oil-in-water emulsions are investigated in this study. Furthermore, there is also limited information on the utilization of the plant extracts incorporated into a film as active packaging. Therefore, this investigation aimed to: (a) investigate the potential antioxidant properties of BL extract using the Trolox Equivalent Antioxidant Capacity (TEAC) assay, and Electron Paramagnetic Resonance (EPR) scavenging activity; (b) demonstrate the ability of lyophilised BL to inhibit lipid deterioration directly in oil-water emulsions; and (c) study the effectiveness of gelatin-based film treated with lyophilised BL in retarding lipid oxidation in meat patties.

## 2. Experimental Section

### 2.1. Plant Material

Commercial dried BL was kindly supplied by Pàmies Hortícoles (Balaguer, Spain), a registered herbal company. All reagents and solvents used were of analytical grade and obtained from Panreac (Barcelona, Spain) and Sigma Aldrich (Gillingham, England).

### 2.2. Extraction of BL Extract

Dried BL was finely ground using a standard kitchen food processor. Ground BL was extracted with 50:50 (*v*/*v*) ethanol:water always in the ratio 1:20 (*w*/*v*). The extractions were performed in the dark at 4 ± 1 °C for 24 h, with constant stirring. The solutions of BL extract were recovered by filtration using Whatman Filter paper, 0.45 µm. Part of the supernatant was taken for subsequent use to determine the antiradical capacity. The remaining supernatant was measured and the excess ethanol was removed under vacuum using a rotary evaporator (BUCHI RE111, Switzerland) and kept frozen at −80 °C for 24 h. All extracts were dried in a freeze dryer (Unicryo MC2L −60 °C, Munich, Germany) under vacuum at −60 °C for 3 days to remove moisture. Finally, lyophilised BL were weighed to determine the soluble concentration (g/L) as described by Zhang *et al.* [14].

### 2.3. Determination of Total Phenolic Compound (TPC)

The Folin–Ciocalteu method was used to determine the total phenolic content as described by Santas *et al.* [15].

### 2.4. Determination of Antioxidant Activity Using TEAC Assay

The antioxidant capacity of BL was measured by a modified TEAC assay as described by Skowyra *et al.* [16], which was based on the method of Miller *et al.* [17].

### 2.5. Determination of Radical Scavenging Activity Assay Using Electron Paramagnetic Resonance (EPR)

EPR radical scavenging activity was measured following the method of Azman *et al.* [18]. BL were extracted with MeOH in a ratio of 1:10 (*w*/*v*) and the soluble concentration of BL was determined as described in the procedure above. A spin-trapping reaction mixture consisted of 100 μL of 5,5-dimethyl-1-pyrroline-*N*-oxide (DMPO) (35 mM), 50 μL of H_2_O_2_ (10 mM), and 50 μL BL extract solution at different concentrations. Ferulic acid was used as reference (0–20 g/L) and the control was pure MeOH. Finally, 50 μL of FeSO_4_ (2 mM) was added to the mixture. The final solutions were transferred to a narrow quartz tube and introduced into the cavity of the EPR spectrometer. The spectrum was recorded for 10 min. X-band EPR spectra were recorded with a Bruker EMX-Plus 10/12 spectrometer (Bruker Española S.A., Madrid, Spain) under the following conditions: microwave frequency, 9.88 GHz; microwave power, 30.27 mW; centre field, 3522.7 G; sweep width, 100 G; receiver gain, 5.02 × 10^4^; modulation frequency, 100 kHz; modulation amplitude, 1.86 G; time constant, 40.96 ms; conversion time, 203.0 ms.

### 2.6. Determination of Antioxidant Activity in Food Model

#### 2.6.1. Removal of Tocopherols from Sunflower Oil

Alumina was placed in an oven at 200 °C for 24 h, and then removed and allowed to cool in a desiccator until it reached room temperature. Sunflower oil was passed twice through the alumina in a column to remove the tocopherols as described by Yoshida *et al.* [19]. Finally, the filtered oil was stored at −80 °C until use.

#### 2.6.2. Preparation of Emulsion

Oil-in-water emulsion was prepared using a method adapted from Azman *et al.* [20]. The final samples were prepared using (1) control (no addition); (2) 0.2 g/kg BHA; or (3) 1 g/kg lyophilised BL. The emulsion for each sample was prepared in quadruplicate, obtaining a total of 12 samples and stored in the dark and allowed to oxidise at 37 °C. The pH of the samples was measured four times for each sample (pH meter GLP21, Crison Instruments, Barcelona, Spain) as a parameter to investigate its correlation with peroxide value (PV).

#### 2.6.3. Determination of Peroxide Value (PV)

The primary oxidation products were measured using peroxide value (PV) according to the thiocyanate method of the Association of Official Analytical Chemists (AOAC) 8195 [21]. Ferrous chloride solution was prepared in hydrochloric acid (1 M) with the addition of iron(II) chloride (2 mM, final concentration). Ammonium thiocyanate solution was prepared in water (2 mM, final concentration). The assay was performed with a drop of emulsion between 0.007 and 0.01 g, diluted with ethanol. From this solution, the required amount of sample, varying according to the degree of oxidation, was transferred into a cuvette and ethanol was added into the sample. Ferrous chloride and ammonium thiocyanate solutions were added, each in a proportion of 1.875% (*v*/*v*), final concentration. The absorbance was measured spectrophotometrically at λ = 500 nm. The results were expressed as meq hydroperoxides/kg of emulsion.

#### 2.6.4. Preparation of Gelatin-Based Film with Antioxidant Coating

The fabrication of gelatin-based film with antioxidant coating was adapted and characterized using the method of Bodini *et al.* [22]. During the cooling of the filmogenic solution after the solubilization of sorbitol, 1 g/kg of BL extract/gelatin was added. Fat and joint tissues were trimmed off lean meat (2000 g) and the meat was minced through 8-mm industrial plates. Then, the meat was moulded to a thickness of 1.5 cm. For each slice, films (5 × 5 cm^2^) were placed on both sides, with either control film (no addition of antioxidant) or BL film (1 g/kg lyophilised BL). Control samples were prepared in the same manner except that the slices were not covered with any film. Subsequently, the samples were packed in polypropylene trays prior to storage at 4 °C for 12 days.

#### 2.6.5. Thiobarbituric Acid Reacting Substances (TBARS)

TBARS measurement was used to measure the extent of lipid oxidation during the storage period as described by Grau *et al.* [23]. Sample (1 g) was weighed in a tube and mixed with 3 ml/L aqueous EDTA. Then, the sample was immediately mixed with 5 mL of thiobarbituric acid reagent using an Ultra-Turrax (IKA, Germany) at 32,000 rpm speed for 2 min. All procedures were carried out in the dark and all samples were kept in ice. The mixture was incubated at 97 ± 1 °C in hot water for 10 min and shaken for 1 min during the process to form a homogeneous mixture. The liquid sample was recovered by filtration (Whatman Filter paper, 0.45 µm) after the sample was cooled for 10 min. The absorbance value for each sample was measured at 531 nm using a spectrophotometer. The TBARS value was calculated from a Malondialdehyde (MDA) standard curve prepared with 1,1,3,3-tetraethoxypropane and analyzed by linear regression. All results were reported as mg malonaldehyde/kg sample.

### 2.7. Statistical Analysis

A one-way analysis of variance (ANOVA) was performed using Minitab 16 software program (Addlink Software Cientifico, Barcelona, Spain) (α = 0.05). The results were presented as mean values (*n* ≥ 3).

## 3. Results and Discussion

### 3.1. Extraction Yield, Total Phenolic Content (TPC) and Antioxidant Activity

The extraction yield, total polyphenols (TPC), and antioxidant activity in extracts of bearberry leaves obtained with 50:50 *v*/*v* ethanol:water are shown in Table 1. On average, 1.6 ± 0.01 g of extract pulp was recovered from 5 g of bearberry extract after 3 days freeze drying (*p* > 0.05).

Pegg *et al.* (2005) reported that the total phenolic content of BL extract was 312 mg/g DW for 95% (*v*/*v*) ethanol extraction [6]. It was much higher than what we reported for 50% (*v*/*v*) ethanol extract. However, the total phenolic content value obtained from the BL water infusion was very low with 160.78 ± 2.84 g/kg sample [24]. The mixtures of alcohol and water was more efficient in extracting phenolic compounds and gave a better yield than water because some phenolic constituents do not dissolve in water. Meanwhile, the antioxidant activity of BL assessed using the TEAC method was 90.42 mmol of TE/g DW. A recent study reported the antioxidant activity of BL in the TEAC assay was 3.19 ± 0.01 mol of TE/kg sample following extraction by an infusion method which is much lower than our value [24]. The antioxidant activity of bearberry leaf achieved in the TEAC assay indicates the potency of the extract to scavenge the radical cation ABTS^•+^ (2,2’-azino-bis(3-ethylbenzothiazoline-6-sulphonic acid)) generated in the assay. The use of several methods allows a more general assessment of the antioxidant properties of the plant. The variations of data were influenced by the sample preparation, type of extraction (solvent, temperature, *etc.*), selection of end-points, and method of expression of the results. Several studies have determined the antioxidant activity of BL extract using *in vitro* analysis. A few studies reported bearberry extract scavenging ability using the DPPH radical and the ability of the extract to reduce ferric(III) ions to ferrous(II) ions using the FRAP (fluorescence recovery after photobleaching) method [15,16,17]. The polyphenol constituents in the extract contribute the most to the antioxidant activity. The infusion of BL showed an abundance of phenolic acid components at trace concentrations including catechin and its derivatives, epigallocatechin gallate, epigallocatechin, and epicatechin. These catechins have a strong antioxidant capacity mainly linked to their radical scavenging activity [18].

### 3.2 Analysis of Free Radical Activity Assays

**Figure 1:**
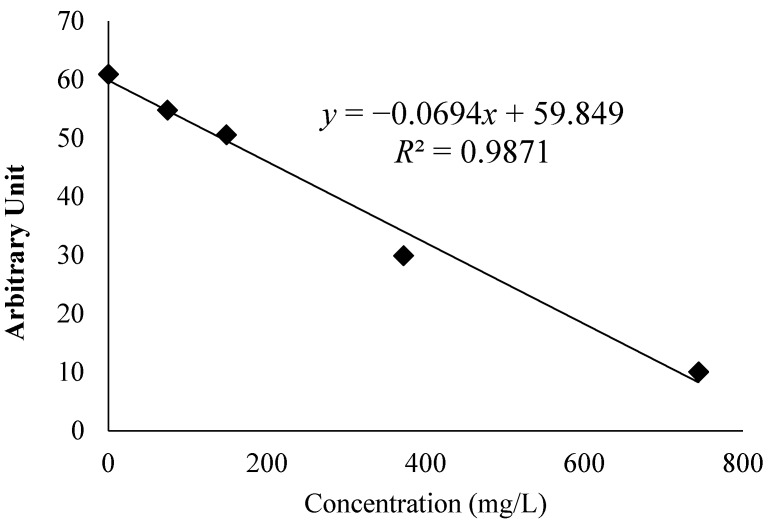
Variation in the area of the electron paramagnetic resonance (EPR) spectra of the radical adduct DMPO–OCH_3_ generated from a solution of H_2_O_2_ (2 mM) and FeSO_4_ (0.04 mM) with DMPO (14 mM) as spin trap in MeOH as solvent. The EPR signal was decreased with the increased of concentration of the BL methanol extracts. The EPR signal decreased at higher antioxidant activity.

In the present study, methanolic extracts from BL were examined by EPR spectroscopy for their capacity to act as radical scavenger towards the methoxy radical (CH_3_O^•^) generated by the Fenton reaction. This method was used to evaluate the scavenging ability of plant extracts for the free methoxy radical (CH_3_O^•^). Figure 1 shows the decrease in the EPR signal with increasing concentration of BL extract. The free radical scavenging activity of the extracts against methoxy (CH_3_O^•^) radical was investigated by a competitive method in the presence of DMPO as spin trap and recorded by the spectrum generated by EPR spectroscopy. The CH_3_O^•^ radical generated by the Fenton procedure has a relatively short half-life, which means it must be identified by EPR as the stable nitroxide adduct with DMPO, DMPO–OCH_3_ (hyperfine splitting constants, a_N_ = 13.9 G and a_H_ = 8.3 G). This stable DMPO–OCH_3_ compound can be quantified by the double integration value of the signal from EPR. The extract containing antioxidants at different concentrations may compete with the spin trap DMPO in the scavenging of methoxy radicals. Thus, the effect decreases the amount of radical adducts and, accordingly, decreases the intensity of the EPR signal. The best fit with intensity of EPR signal exhibited a linear function (Figure 1), given by Equation (1):

*y* = −0.0694*x* + 59.849; *R^2^* = 0.9871 (1)

where the *x* values were in mg/L.

The graph indicates the exponential value of the signal of the spectrum decreased as the amount of bearberry extract increased. Azman *et al.* demonstrated the scavenging ability of catechins with methoxy radical using this assay [18]. These catechins were also found in bearberry extract by Valjkovic *et al.* and these compounds contributed to the ability to scavenge methoxy radical in this assay [24]. Furthermore, BL scavenging ability has been previously reported by Amarowicz *et al.* using hydroxyl free radicals (HO^•^) measured by EPR [11].

### 3.3. Antioxidant Effects in Stored o/w Emulsion

Methods have been developed to understand the effect of natural antioxidants in model foods such as emulsions and active film packaging. Adding natural antioxidants to food not only delays the oxidation process but also enhances the nutritional quality of the food through direct ingestion. In previous work, the effect of bearberry leaf extract in oil-water emulsion has not been described. A model emulsion was used to assess the deterioration of lipids at two stages of oxidation, which were the primary oxidation products (Peroxide Value) and the secondary oxidation products (TBARS). In addition, the change in pH was monitored, since pH tends to fall during oxidation.

**Figure 2:**
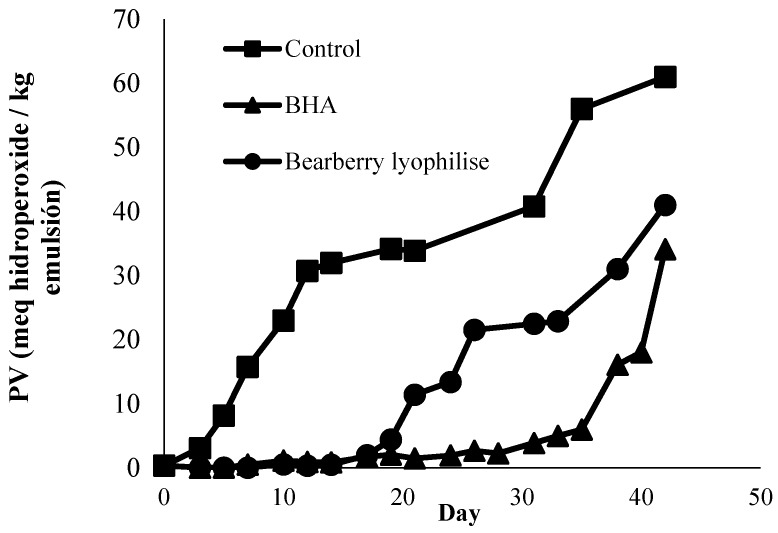
Change in peroxide value over time stored at 37 °C. Each value is expressed as mean (*n* = 3).

The development of primary oxidation products was monitored by the evaluation of hydroperoxide formation (PV) during storage (Figure 2). Primary degradation of lipids measured by PV occurs due to the reaction between oxygen and unsaturated fatty acids thatform hydroperoxides. The induction time is defined as the time for samples to reach 10 meq hydroperoxides/kg of emulsion. This value can be used as a measure of the stability of emulsions. The limits of oxidation products in fat products (animal, plant and anhydrous) including margarine and fat preparations were set at <10 meq hydroperoxides/kg as a guarantee of the product quality [20]. When the peroxide value of the sample is greater than 10 meq hydroperoxide/kg, the sample is in a highly oxidised state and starts to become rancid. The PV value of the control emulsion increased rapidly, reaching more than 10 meq hydroperoxide/kg after only 6 days (*p* < 0.05). The sample containing BL 1 g/kg reached the end of the induction time after 20 days while the BHT samples reached this state after 36 days of storage. Several studies investigated the effects of adding natural antioxidants to delay the lipid deterioration in food model emulsions. Skowyra *et al.* [16] found that an emulsion containing 48 μg/mL of Tara extract took 13 days to reach more than 10 meq hydroperoxide/kg, and Roedig–Penman and Gordon [25] reported that an emulsion containing green tea extract required eight days to reach the end of the induction time. Emulsion containing 100 mg/L of rosemary and thyme extract displayed low PV which remained below 10 for 25 days of storage [25].

**Figure 3:**
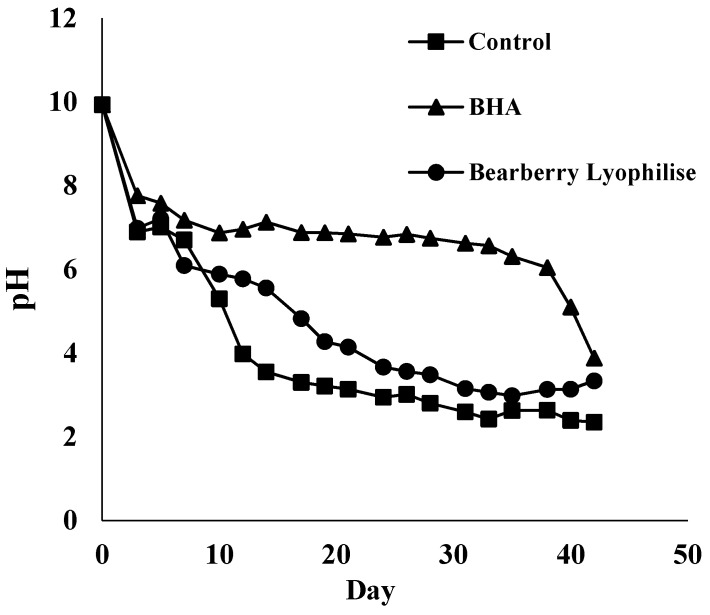
Change of pH over time stored at 37 °C. Each value is expressed as mean (*n* = 3).

Primary oxidation occurs rapidly in the fat phase of the product due to the formation of highly unstable hydroperoxides that break down easily. This process results in the formation of ketones, epoxides, or organic acids which are acidic, and leads to changes in the pH [16]. Figure 3 shows that the pH value dropped over time, and this change was inversely proportional to the increase of PV. By comparison, emulsion containing BHA showed a significant pH difference from the pH values for BL and the control sample (*p* < 0.05). BL samples remained higher than pH 6 for 10 days of storage and the pH declined gradually until 40 days.

A number of researchers suggested a positive effect of pH on oxidation rate which was influenced by natural antioxidants [16,17,26]. Pehlivan *et al.* [27] reported that edible vegetable oils contained heavy metals such as iron up to 0.2 mg/kg oil. The levels of some metal compounds, if high enough, will promote oxidation which affects the pH [28]. Furthermore, the redox state of metals and the activity, solubility, stability, and chelation capacity of antioxidants are among the parameters that affect the rate of change of pH in oil emulsions [29].

**Figure 4:**
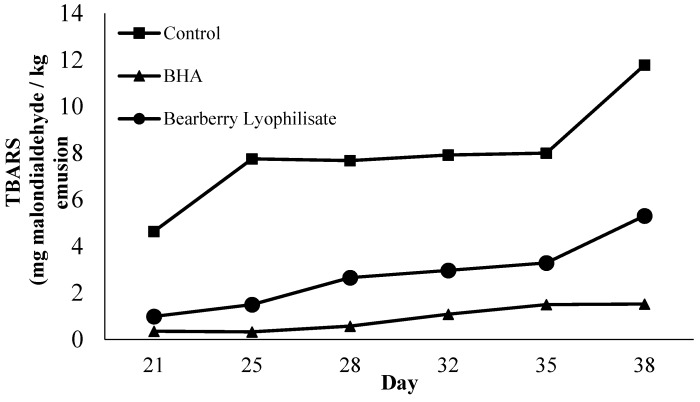
Change of TBARS over time during storage at 37 °C. Each value is expressed as mean (*n* = 3).

Analysis of oxidation in the emulsions was extended by measuring a secondary oxidation product in the emulsion using the TBARS method (Figure 4). Malondialdehyde (MDA) compounds produced are responsible for the alteration of flavor, rancid odor, and the undesirable taste in foods [30]. The acceptable limits of TBARS value in fat products was set at 1.0 mg malondialdehyde/kg [31]. Figure 4 shows the secondary oxidation products monitored by TBARS assay. Control sample experienced TBARS value of greater than 4.0 mg malondialdehyde/kg (*p* < 0.05) on the 3 weeks storages compare to samples treated with antioxidant with value of TBARS that less than 1 mg malondialdehyde/kg. Over the 25 days of storage, samples with antioxidants had a TBARS value of approximately 1 mg malondialdehyde/kg sample, which may not change the physical and visual properties of the product. Emulsion treated with BHA exhibited the lowest TBARS value throughout the storage period and samples with bearberry had TBARS values of lower than 2 mg malondialdehyde/kg (*p* < 0.05). 

Many findings demonstrated that the efficiency of natural plant extracts as antioxidants in various concentrations applied possibly depends on the variation in the system tested and initial plant extraction. The active properties of BL was reported several times by Amarowicz *et al.* [11,32]. Quercetin, one of the main compounds found in the BL extract, showed positive effects against lipid deterioration in oil-in-water emulsion [9]. The leaves consist of an abundance of phenolic compounds, which may contributed to the antioxidant properties, reducing power, and antiradical properties as described in much of the literature [7,11,32]. The antioxidant activity of phenolic linked with phenolic compounds is related to the hydroxyl group linked to the aromatic ring, which is capable of donating hydrogen atoms and neutralizing free radicals. This mechanism prevents further propagation of lipid oxidation, which can be measured by the TBARS method [33]. The behavior of BL as a natural antioxidant in a food model was reported in the literature [12,34]. Pegg *et al.* (2005) [9] reported 200 ppm of bearberry extract inhibited TBARS formation in cooked pork patties after seven days of storage. Meanwhile, Carpenter *et al.* [12] demonstrated that the addition of BL impeded lipid oxidation in raw pork patties for 9–12 days of refrigerated storage relative to control. To the best of our knowledge, this is the first report of the inhibitory effect of BL using oil-in-water emulsion.

### 3.4. Antioxidant Effects in Active Film Packaging with Bearberry Coating

**Figure 5:**
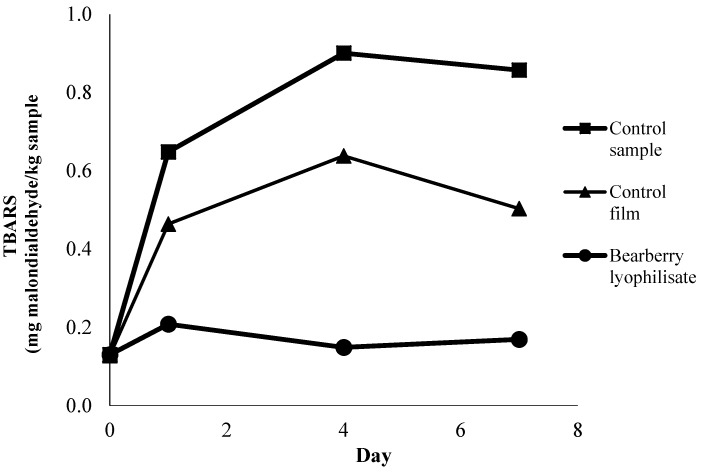
Changes in TBARS values (mg malondialdehyde/kg sample) of control and sample containing BL extract during seven days of storage at 4 ± 1 °C without light. Each sample was measured in triplicate and the average standard deviation for each sample was less than 5%.

The TBARS index (Figure 5) revealed that secondary oxidation of the control beef patties increased progressively as storage advanced. Meat patties without any film on the patties (control sample) experienced the highest TBARS values and the values were significantly different than those of all other samples. Coating with gelatin-based film enriched with BL extract lowered the oxidation rate significantly throughout the storage period (*p* < 0.05). At the end of the storage time, patties stored under the gelatin film with BL contained only 0.17 mg malondialdehyde/kg sample compared to the control TBARS value of 0.86 mg malondialdehyde/kg sample. There are very few reports dealing with the effects of edible gelatin-based films containing natural plant extracts. Published studies of gelatin-based films focused on the physical, chemical, and mechanical properties of the film [20,21,22,23,24,25,26,27]. However, there are only a few studies reported the antioxidant effects of gelatin film treated with various natural antioxidants. Recently, several reports proposed that the combination of natural plant with antioxidant with gelatin film not only to improve its physical properties also delays the oxidation process within the food packaging [34,35]. This study reported the preliminary results of the effects of bearberry extract incorporated into gelatin film which successfully delayed oxidation in a muscle food. 

## 4. Conclusions

In conclusion, BL extract showed a positive effect due to its antioxidant activity and scavenging ability, which delayed lipid oxidation in an emulsion and when used as an active component in gelatin film packaging. BL extract contained a high concentration of phenolic compounds and good antioxidant activity when assessed by total phenolic content and TEAC assay, respectively. The methanol extract showed scavenging ability against methoxy radicals generated by the Fenton reaction when assessed by EPR. Lyophilised BL (0.1% *w*/*w*) was applied as an antioxidant in an emulsion food model and significantly inhibited lipid oxidation during 20 days of storage. A preliminary study of the effect of gelatin-based film coated with BL extract showed that it significantly delayed degradation of lipids in meat patties (*p* < 0.05). Therefore, this study confirmed that bearberry leaves can be used as a source of antioxidants with potential use for the food industry.

## Figures and Tables

**Table 1:** Extraction yield, polyphenol content and antioxidant activity of bearberry leaf extracts.

Activity Bearberry Extract	Extraction Solvent 50:50 (*v*/*v*) EtOH:H_2_O *
Extraction yield (%)	32.1% ± 0.03%
Total phenolic content (mg GAE/g DW)	102.11 ± 7.12
TEAC (mmol of TE/g DW)	90.42 ± 1.83

*: Results are expressed as mean ± standard deviation (*n* = 3). Gallic acid equivalent (GAE), Trolox equivalent antioxidant activity (TEAC), Trolox equivalent (TE), dry weight (DW).
